# Toxicity of the Herbicide Atrazine: Effects on Lipid Peroxidation and Activities of Antioxidant Enzymes in the Freshwater Fish *Channa Punctatus* (Bloch)

**DOI:** 10.3390/ijerph7083298

**Published:** 2010-08-24

**Authors:** Christopher Ddidigwu Nwani, Wazir Singh Lakra, Naresh Sahebrao Nagpure, Ravindra Kumar, Basdeo Kushwaha, Satish Kumar Srivastava

**Affiliations:** 1 Department of Applied Biology, Ebonyi State University, PMB 053, Abakaliki, Ebonyi State, Nigeria; 2 National Bureau of Fish Genetic Resources (Indian Council of Agricultural Research), Canal Ring Road, PO-Dilkusha Lucknow 226 002 (UP), India; E-Mails: lakraws@hotmail.com (W.S.L.); nagpurens@yahoo.com (N.S.N.); ravindrakumar@scientist.com (R.K.); basdeokushwaha@yahoo.co.in (B.K.); satishsrivastava@mail.com (S.K.S.)

**Keywords:** atrazine, toxicity, C. punctatus, oxidative stress, environmental pollution, LC_10–90_

## Abstract

The present study was undertaken to evaluate the toxicity and effects of a commercial formulation of the herbicide atrazine (Rasayanzine) on lipid peroxidation and antioxidant enzyme system in the freshwater air breathing fish *Channa punctatus*. The 12, 24, 48, 72 and 96 h LC_50_ of atrazine, calculated by probit analysis, were determined to be 77.091, 64.053, 49.100, 44.412 and 42.381 mg·L^−1^, respectively, in a semi static system with significant difference (p < 0.05) in LC_10–90_ values obtained for different times of exposure. In addition to concentration and time dependent decrease in mortality rate, stress signs in the form of behavioral changes were also observed in response to the test chemical. In fish exposed for 15 days to different sublethal concentrations of the herbicide (1/4 LC_50_ = ∼10.600 mg·L^−1^, 1/8 LC_50_ = ∼5.300 mg·L^−1^ and 1/10 LC_50_ = ∼4.238 mg·L^−1^) induction of oxidative stress in the liver was evidence by increased lipid peroxidation levels. The antioxidants superoxide dismutase (SOD), catalase (CAT) and glutathione reductase (GR) responded positively in a concentration dependent pattern, thus, suggesting the use of these antioxidants as potential biomarkers of toxicity associated with contaminations exposure in freshwater fishes.

## 1. Introduction

The use of herbicides to control weeds has been recognized as a part of agricultural practices throughout the world. Unfortunately, the indiscriminate use of these herbicides to improve agricultural production and yield may have impacts on non-target organisms, especially aquatic life forms and their environment. Atrazine (2-chloro-4-ethylamino-6-isopropylamino-*s*-triazine) is one of the most commonly used herbicides found in the rural environments. It is extensively used on corn, sorghum, sugarcane, pineapples, and to some extent on landscape vegetation. Rated as moderately toxic to aquatic species, atrazine is mobile in the environment and is among the most detected pesticides in streams, rivers, ponds, reservoirs and ground waters [1–3]. It has a hydrolysis half-life of 30 days and relatively high water solubility (32 mg·L^−1^), which aids in its infiltration into ground water [4]. Atrazine concentrations of 20 μg·L^−1^ have been commonly detected in surface water runoff, while concentrations as high as 700 μg·L^−1^ have been reported [5–7]. Due to the low persistence of atrazine herbicide, repeated applications are practiced for the control of weeds in agricultural fields, and as a result, large quantities of the herbicide find their ways into water bodies. The mode of action of atrazine is blocking electron transport in photosystem 11 leading to chlorophyll destruction and blocking photosynthesis. When atrazine was first released for agricultural use, it was thought that since photosynthesis is limited to plants, animals would be immune to any effects of atrazine. It was soon suspected that atrazine might have non-target action in animals including genotoxic [8], clastogenic [9] and biochemical effects [10]. The indiscriminate use of this herbicide, careless handling, accidental spillage or discharge of untreated effluents into natural water ways have harmful effects on the fish populations and other aquatic organisms and may contribute to long term effects in the environment. Sublethal effects with biochemical and histopathological alteration of fish tissues may occur with long term exposure to levels of less than 2 mg·L^−1^ of atrazine [11].

Fish can serve as bio-indicators of environmental pollution and can play significant roles in assessing potential risk associated with contamination in aquatic environment since they are directly exposed to chemicals resulting from agricultural production via surface run-off or indirectly through food chain of ecosystem [12]. Fish are endowed with defensive mechanisms to counteract the impact of reactive oxygen species (ROS) resulting from metabolism of various chemicals or xenobiotics. Oxidative stress develops when there is an imbalance between pro-oxidants and antioxidants ratio, leading to the generation of ROS. Environmental contaminants such as herbicides, heavy metals and insecticides are known to modulate antioxidant defensive systems and to cause oxidative damage in aquatic organisms by ROS production [13–16]. ROS such as hydrogen peroxide (H_2_O_2_), superoxide anion O_2_^−^ and hydroxyl radical (OH^·^) at supranormal levels can react with biological macromolecules potentially leading to enzyme inactivation, lipid peroxidation (LPO), DNA damage and even cell death [17,18] but at low concentrations their effects are less pronounced.

Several ecotoxicological characteristics of *C. punctatus* such as wide distribution in the freshwater environment, availability throughout the year, easy acclimatization to laboratory conditions and commercial importance make this species an excellent test species for toxicity and biochemical studies [19]. There have been studies on toxicities of atrazine-based herbicides; however meager information is available about their toxicity and effects on fish and aquatic organisms especially on sub-chronic exposure. Determination of the toxicity is essential for determining sensitivity of the animals to the toxicants and also useful for evaluating the degree of damage to the target organs and the consequent physiological, biochemical and behavioral disorders. Thus, to supplement risk assessment studies of these herbicides, it is important to obtain information on their toxicity and effects on some local species like *C. punctatus*.

The aim of the present study was to assess the toxicity of a commercial formulation of the herbicide atrazine (Rasayanzine) and the impact on some biochemical indices in *C. punctatus*. The information obtained may be useful for management and monitoring of atrazine (Rasayanzine) contamination in the environment.

## 2. Materials and Methods

### 2.1. Experimental Fish Specimens and Chemicals

Freshwater air-breathing fish C. punctatus (Bloch; Family: Channidae, Order: Perciformes) were procured from local sources. The specimens had an average weight and length of 14.40 ± 1.00 g and 10.42 ± 1.02 cm, respectively. Fish specimens were subjected to a prophylactic treatment by bathing twice in 0.05% potassium permanganate (KMnO_4_) for two min. to avoid any dermal infections. The fishes were then acclimatized for two weeks under laboratory conditions in semi-static systems. They were fed boiled eggs, minced goat liver and poultry waste materials. The fecal matter and other waste materials were siphoned off daily to reduce ammonia content in water. For the present study commercial formulations of atrazine (50% WP) with the trade name “Rasayanzine”, manufactured by Krishi Rasayan Exports Ltd (India) was purchased from the market.

### 2.2. Determination of Sub-Lethal Concentrations

Acute toxicity bioassays to determine the 96 h LC_50_ value of atrazine was conducted in a semi-static laboratory system according to the OECD guideline No 203 [20]. Atrazine was dissolved in filtered distilled water and added to the aquarium following the method of Pluta [21]. The water with herbicide was changed after every 24 h by adding the fresh atrazine solution in order to counterbalance decreasing herbicide concentrations because of its hydrolysis in water. The experiment was conducted in glass aquaria (60 × 30 × 30 cm), containing 40 L of dechlorinated and gentle aerated tap water. A set of 10 fish specimens were randomly exposed to each of the nominal atrazine concentrations (25.0, 32.0, 39.0, 46.0, 53.0 and 60.0 mg·L^−1^) and a control (0.00 mg·L^−l^) and the experiment was set in triplicate to obtain the LC_50_ values of the test herbicide for the species. The LC_50_ value of atrazine for *C. punctatus* was determined as 42.381 mg·L^−1^ following the probit analysis method as described by Finney [22]. Based on the 96 hLC_50_ values, the fish were then exposed for 15 days to three sub-lethal concentrations of the herbicide (1/4 LC_50_ = ∼10.600 mg·L^−1^, 1/8 LC_50_ = ∼5.300 mg·L^−1^ and 1/10 LC_50_ = ∼4.238 mg·L^−1^) and used for the enzyme assays. A set of 10 fish were also simultaneously maintained in tap water (0.00 mg·L^−1^) as the control. No mortalities occurred following the exposure to the three sublethal concentrations of the herbicide during the experiment.

### 2.3. Tissue Sampling

The fish specimens were exposed to the three aforementioned test concentrations of atrazine along with the control and the experiments continued for 15 days. At the end of every 1, 5, 7, 10 and 15 days five fishes were sacrificed by cervical decapitation. The liver was dissected out carefully, washed in ice-cold 1.15% KCl solution, blotted and weighed. They were then homogenized in homogenizing buffer (50mM Tris-HCl mixed with 1.15 KCL and pH adjusted to 7.4) using a motor-driven Teflon Potter–Elvejhem homogenizer. The resulting homogenate was centrifuged at 10,000 g for 20 min in a refrigerated centrifuge at 4 °C. The clear supernatants collected were used for protein estimation and assaying the activity of enzymes.

### 2.4. Biochemical Assays

Lipid peroxidation was assayed by measuring malondialdehyde (MDA) formation as described by Sharma and Krishnamurthy [23]. Briefly, 1.0 mL of homogenate prepared in KCl solution was incubated at 37 °C for 30 min. Proteins were precipitated by adding 1 mL of 10% trichloroacetic acid (TCA) and then centrifuged at 2,000 g for 15 min. One mL of supernatant was taken as an aliquot in separate tube to which 1 mL of thiobarbituric acid reacting substances (TBA) solution was added. The tubes were kept in boiling water bath for 10 min. After cooling the tubes, the optical density was read at 535 nm. Superoxide dismutase (SOD) activity was determined by measuring the inhibition of autoxidation of adrenaline at pH 10.2 at 30 °C as described by Misra and Fridovich [24]. Activity of catalase (CAT) was determined according the procedure of Clairborne [25] by following the absorbance of H_2_O_2_ at 240 nm, pH 7.0 and 25 ºC. Activity of glutathione reductase (GR) was measured according to the method of Massey and Williams [26]. All assays were run in the triplicate. The protein content of the various fractions was estimated by the Folin-Phenol reaction method as described by Lowry *et al*. [27].

### 2.5. Data Analysis

The data obtained were statistically analyzed by statistical package SPSS (version 16). The data were subjected to one way ANOVA and Duncan’s multiple range test was used to determine the significant difference between the means at 5% probability level. The safe level estimates of the herbicides were drawn based on Sprague [28], CWQC [29], NAS/NAE [30], IJC [31], CCREM [32] and Hart *et al*. [33].

## 3. Results

### 3.1. Physico-Chemical Parameters of the Test Water

The physico-chemical characteristics of the test water are presented in (Table 1). The water temperature varied from 24.20 to 25.40 °C and the pH ranged from 7.4 to 7.9. The dissolved oxygen concentration ranged from 6.7 to 7.8 mg·L^−1^; conductivity values ranged from 260 to 300 μM·cm^−1^ while total hardness varied from 200–230 mg·L^−1^ during the experimental period.

### 3.2. Toxic Stress and Poisoning Symptoms in Fish

Fish subjected to different concentrations of atrazine herbicide displayed uncoordinated behavior. At the initial exposure, fish were alert, stopped swimming and remained static in position in response to the sudden changes in the surrounding environment. After some time they tried to avoid the toxic water with fast swimming and jumping. Faster opercula activity was observed as surfacing and gulping for air. In tanks with higher concentrations of test herbicide, the fish swam erratically with vigorous jerks of the body. Their fins became hard and stretched due to stretching of body muscles. They secreted copious amounts of mucus from whole body continuously and soon a thick layer of mucus was found deposited in the buccal cavity and gills. Body pigmentation was decreased. Ultimately fish lost their balance, consciousness, engage in rolling movement and became exhausted and lethargic. Lastly, they remained in vertical position for a few minutes with anterior side or terminal mouth up near the surface of the water, trying to gulp air and tail in a downward direction. Soon they settled at the bottom of the tank, and after some time their bellies turned upward and the fish died.

### 3.3. Median Lethal Concentration (LC_50_) and Application Factor

Median lethal concentration (LC_50_) is the most widely accepted basis for acute toxicity test and it is the concentration of a test chemical which kills 50% of the test organisms after a particular length of exposure, usually 96 h. Generally in toxicity tests, death is a decisive criterion because it is easy to determine and has obvious biological and ecological significance. The LC_50_ values (with 95% confidence limits) of different concentrations of atrazine (Table 2) were found to be 77.091, 64.053, 49.100, 44.412 and 44.381 mg·L^−1^ for 12, 24, 48, 72 and 96 h LC_50,_ respectively following Finney’s [22] method and using SPSS (version 16). A dose dependent increase and time dependent decrease were observed in mortality rate, such that as the exposure time increases from 12 to 96 h, the median concentration was reduced. There were significant differences (p < 0.05) in LC_10–90_ values obtained for different times of exposures. It was observed that as the concentration of the herbicide increased fish mortality also increased that indicates a direct proportional relationship between mortality and concentration of atrazine herbicide. No mortality was observed in the control during the experimental period. There were variations in safe level estimated by different methods at 96 h of exposure of the fish as presented in (Table 3).

### 3.4. Oxidative Stress Marker

Lipid peroxidation determined by the formation of TBARS is one of the commonly used markers of oxidative stress. The effects of different concentrations of atrazine on TBARS formation in the liver of *C. punctatus* are presented in Table 4. A significant effect of both concentrations and duration of exposure (p < 0.05) was observed in the specimen exposed to atrazine. The lowest TBARS formation was observed on the first day of exposure to 4.238 mg·L^−1^of herbicide and there was gradual non linear increase in TBARS formation in the liver tissue with the progression of the experiment, with the highest TBARS formation on the day 15. At the highest sublethal concentration of atrazine (10.600 mg·L^−1^), TBARS formation increased by a factor of 1.450 on day 1 and by 4.431 on day 15, when compared with the control.

### 3.5. Antioxidant Enzyme Activity

The activities of the enzyme SOD in the liver of the fishes exposed to atrazine herbicide are presented in Table 5. SOD activity was significantly (p < 0.05) elevated in the liver at all the concentrations of atrazine in the experimental group.

SOD activity increased in concentration and time dependent pattern with percentage increase of 28% and 101% observed at the highest concentration (10.600 mg·L^−1^) on day 1 and 15 of the herbicide exposure respectively, as compared to control. The activities of CAT follow the same pattern as SOD in the liver of *C. punctatus* exposed to atrazine but up to day 7 of exposure. CAT activity in the liver was significantly different from the control (p < 0.05) and decreased from 79% on day 7 to 27% on day 15 following exposure to 10.600 mg·L^−1^of atrazine (Table 6).

There was no significant increase (p > 0.05) in GR activity in the liver of the fish exposed to sublethal concentration of the herbicide compared with the control except on day 10 and 15. GR activity increased significantly to 44% in the liver on day 10 to 73% on day 15 following exposure to 10.600 mg·L^−1^ of the herbicide (Table 7).

## 4. Discussion

Fish are often used as sentinel organisms for ecotoxicological studies because they play number of roles in the trophic web, accumulate toxic substances and respond to low concentration of mutagens [34] therefore, the use of fish biomarkers as indices of the effects of pollution are of increasing importance and can permit early detection of aquatic environmental problems [35,36].

Acute toxicity data has been used to derive water quality guidelines for regulatory measures [37]. The result of the LC_50_ (median lethal concentration) for atrazine in the present study at 96 h was 42.381 mg·L^−1^. The results show that the toxicity of atrazine for *C. puctatus* is both time and concentration dependent, thus, accounting for differences in LC_10–90_ values obtained at different concentrations and time of exposure. However, some other researchers have shown that exposure time is not significant in LC_50_ determinations for fish [38]. The LC_50_ value obtained for *C. puctatus* in this study is higher than that reported by Bathe *et al*. [39]; Neškovic *et al*. [11] and Hussein *et al*. [40], who reported LC_50_ vales of 16.0, 18.8 and 9.37 mgl^−1^ for *Lepomis macrochirus* (Bluegill sunfish), *Cyprinus carpio* and *Oreochromis niloticus*, respectively, exposed to atrazine. Toxicity of chemicals to aquatic organisms has been shown to be affected by age, size and health of the species [41]. Physiological parameters like quality, temperature, pH, dissolved oxygen and turbidity of water, amount and kind of aquatic vegetation, concentration and formulation of chemical and its exposure also greatly influence such studies [42,43]. Sprague [44] observed variability in acute toxicity even in single species and single toxicant depending on size, age and condition of the test species along with experimental factors. The estimated safe level as calculated by multiplying the LC_50_ with application factor (AF) varied from 4.24 × 10^−4^ to 8.657. However, the large variation in safe levels determined by different methods has resulted in controversy over its acceptability [19,45]. Kenega [46] emphasized that the major weakness in calculation of accumulation factor (AF) is its dependence on LC_50_. Warren [47] and Pandey *et al*. [48] had reported that the introduction of toxicant into an aquatic system might decrease the dissolved oxygen concentration which will impair respiration leading to asphyxiation. Fish exposed to atrazine were stressed progressively with time before death. The respiratory impairment due to the toxic effect of atrazine on the gills of *C. punctatus* is similar to the reports of Abdul-Farah *et al*. [41]; De Mel and Pathiratne [49]; Tilak *et al*. [50] and Ayoola [51] that pesticides impair respiratory organs. Death could have, therefore, occurred either by direct poisoning or indirectly by making the medium unconducive for the fish or even by both. The abnormal behavior observed during the exposure period like incessant jumping, restlessness, surface to bottom movement, vigorous jerks were similar to the observations of Hussein *et al*. [40]; Pandey *et al*. [19] and Chandra [52].

The elevated level of lipid peroxidation in the liver of *C. punctatus* in response to the exposure to atrazine as observed in the present investigation suggests that there is increased production of ROS. Increased ROS production may, thus, be associated with the metabolism of atrazine herbicide leading to the peroxidation of membrane lipids of the liver. The liver is noted as site of multiple oxidative reactions and maximal free radical generation [53–55]. Previous investigations have reported the induction of LPO by pesticides such as deltamethrin [56,57], alachlor [58], malathion [52] and butachlor [59] in fish. Lushchak *et al*. [60], however, did not record elevation of lipid peroxidation in the brain and liver of goldfish *Crassius auratus* exposed to sublethal concentration of Roundup^®^. The different responses probably are functions of species, the time of exposure, type and concentration of stressors. The observed LPO resulting from ROS generated by the atrazine may lead to cell apoptosis. ROS and oxidative stress have been demonstrated to be triggers of apoptosis [61]. Oxidative stress may also be due to the depletion of cellular GSH content below the critical level which prevents the conjugation of xenobiotics like atrazine to GSH and thus enables them to freely combine covalently with cell proteins [62]. However, organisms are equipped with interdependent cascades of enzymes to alleviate oxidative stress and repair damaged macromolecules, produced during normal metabolism or due to exposure to xenobiotics. In this cascade, SOD and CAT are the major enzymes in eliminating ROS formed during bioactivation of xenobiotics in the hepatic tissues [63] and the induction of SOD/CAT system provides a first line of defense against ROS. SOD help to dismutase superoxide radical O_2_^−^ to hydrogen peroxide (H_2_O_2_). The increase in CAT activities in the liver as observed in the present study may be in response to H_2_O_2_ produced by SOD activity since CAT is responsible for the detoxification of H_2_O_2_ to water. Enhancement of CAT activity was observed in Cichlid fishes from polluted waters [64], in mullets [65], *Lepomis macrochirus* [66] and *Prochilobus lineatus* [67] exposed to herbicides. CAT activity, however, decreased after day 7 of exposure to atrazine, although the values obtained were significantly (p < 0.05) higher than control. Decrease in CAT activity after the 7th day of exposure could be due to decrease in the rate of reaction as a result of the excess production of H_2_O_2_. The inhibition of CAT by the superoxide radical has been reported [68,69]. In all, the increase in LPO, CAT and SOD activities in the liver tissue as reported in the present investigation supports the hypothesis that sublethal concentrations of atrazine induce oxidative stress in *C. punctatus* and could be an adaptive response to protect the fish from the atrazine-induced free radical toxicity. GR activity was unchanged in the liver of *C. punctatus* following exposure to different concentrations of atrazine, except on day 10 and 15. Similar to the present observation, the hepatic GR activity in the liver of bluegill sunfish (*Lepomis macrochirus*) was not affected following exposure to atrazine for 96 h [66]. It, thus, appears that dose and exposure time affected the activity of GR in *C.* punctatus exposed to atrazine. The elevated GR observed on day 10 and 15 could be part of the protective response in the tissue which suggest that GR pathways could efficiently be used to regulate the ROS formation during the contaminated period. This compensatory response accompanied with the induction of other antioxidants (SOD and CAT) evidently helps to prevent accumulation of free radicals and their products in stressed organisms. The result of these studies evidence that the biochemical responses are dependent on stressor type, species and exposure time. Furthermore, the herbicide may lead to the occurrence of transformation products in water with a potential or actual similar or higher toxicity than their parent [70]. Metabolites/breakdown products of atrazine potentially could have affected the biochemical measurements in exposed *C. punctatus*. Thus further studies are required to determine these biochemical changes after the exposure of fish to atrazine and its transformation products.

## 5. Conclusions

The present investigation indicated that the herbicide atrazine is toxic to fish and further substantiate earlier findings that antioxidant enzymes such as SOD, CAT and GR and LPO in fish could be effectively used as biomarkers of herbicide toxicity.

## Figures and Tables

**Table 1.** Physico-chemical properties of the test water.

**Characteristics**	**Unit**	**Mean**	**Range**
Air temperature	°C	26.20	25.6–26.6
Water temperature	°C	24.40	24.2–25.4
pH	-	7.6	7.4–7.9
Dissolved oxygen	mg·L^−1^	6.8	6.7–7.8
Conductivity	μMcm^−1^	282	260–300
Total hardness	mg·L^−1^	220	200–230

**Table 2.** Lethal concentrations (LC_10–90_) of atrazine depending on exposure time (12–96 h) for *C*. punctatus.

**Point**	**Concentrations (mg·L^−1^) at various exposure times. (95% confidence intervals)**				

	**12 h**	**24 h**	**48 h**	**72 h**	**96 h**
**LC_10_**	50.501^a^ (43.110–56.190)	43.042^b^ (36.661–46.872)	37.383^c^ (33.422–40.111)	34.664^d^ (31.322–37.061)	33.192 ^d^ (29.920–35.542)
**LC_20_**	58.502^a^ (52.680–77.012)	49.331^b^ (442.92–53.272)	41.054^c^ (37.781–43.473)	37.742^d^ (34.890–39.901)	36.093^d^ (33.292–38.201)
**LC_30_**	65.042^a^ (57.474–101.282)	54.443^b^ (50.263–61.351)	43.911^c^ (41.111–46.253)	40.133^d^ (37.622–42.182)	38.343^d^ (35.872–40.344)
**LC_40_**	71.203^a^ (61.291–129.286)	59.210^b^ (54.284–70.050)	46.522^c^ (43.993–48.982)	42.282^d^ (40.015–44.350)	40.382^d^ (38.114–42.306)
**LC_50_**	77.091^a^ (64.816–162.927)	64.053^b^ (57.806–79.92)	49.100^c^ (46.623–51.914)	44.412^d^ (42.252–46.623)	42.381^d^ (40.218–44.406)
**LC_60_**	84.342^a^ (68.503–205.719)	69.293^b^ (61.425–91.522)	51.814^c^ (49.210–55.324)	46.642^d^ (44.417–49.109)	44.471^d^ (42.420–46.822)
**LC_70_**	92.343^a^ (72.620–264.422)	75.372^b^ (65.319–106.103)	54.892^c^ (51.890–59.247)	49.152^d^ (46.801–52.228)	46.834^d^ (44.632–49.607)
**LC_80_**	102.661^a^ (77.612–354.807)	83.164^b^ (70.221–126.014)	58.723^c^ (55.032–64.911)	52.260^d^ (49.501–56.331)	49.754^d^ (47.223–53.412)
**LC_90_**	118.932^a^ (85.017–534.116)	95.321^b^ (77.411–160.716)	64.483^c^ (59.511–73.515)	56.902^d^ (53.322–62.722)	54.113^d^ (50.804–59.332)

Note: Lethal concentration values in rows with different letters significantly differ at p < 0.05.

**Table 3.** Estimate of safe level of atrazine at 96 h exposure time in *C. punctatus*.

**96 h LC_50_ (mg·L^−1^)**	**Method**	**AF**	**Safe level (mg·L^−1^)**
42.38	Hart *et al*. [33]*	–	8.657
	Sprague [28]	0.1	4.240
	CWQC [29]	0.01	0.424
	NAS/NAE [30]	0.1–0.00001	4.24–4.24x10^−4^
	CCREM [32]	0.05	2.129
	IJC [31]	5% of 96hLC_50_	2.129

* C = 48 h LC_50_ × 0.03/S^2^, where C is the presumable harmless concentration and S = 24 h LC_50_/48 h LC_50_

**Table 4.** Formation of thiobarbituric acid reactive substances (TBARS) (nmol/mg protein) in liver of *C. punctatus* exposed to sublethal doses of atrazine for 1–15 days.

**Conc. mg·L^−1^**	**Exposure time (days)**				
	**1**	**5**	**7**	**10**	**15**
00	1.011 ± 0.06^aA^	1.042 ± 0.07^aA^	1.034 ± 0.05^aA^	1.011 ± 0.07^aA^	1.002 ± 0.01^aA^
4.238	1.060 ± 0.03^aA^	1.193 ± 0.05^aAB^	1.351 ± 0.08^aAB^	2.312 ± 0.16^cB^	2.881 ± 0.10^dB^
5.300	1.183 ± 0.45^aA^	1.660 ± 0.19^aB^	2.334 ± 0.16^bC^	2.512 ± 0.13^bB^	3.664 ± 0.17^cC^
10.600	1.472 ± 0.43^aB^	2.884 ± 0.11^bC^	3.022 ± 0.10^bD^	3.064 ± 0.11^bC^	4.431 ± 0.33^cC^

Note: Data are presented as mean ±SE of five fishes in each group, values with different alphabet superscript differ significantly (p < 0.05) between durations within concentration. Values with capital alphabet superscript differ significantly (p < 0.05) between concentrations within duration.

**Table 5.** Activity of Superoxide dismutase (unit/mg protein) in the liver of *C. punctatus* exposed to sub-lethal doses of Atrazine for 1–15 days.

**Conc. mg·L^−1^**	**Exposure time (days)**				
	**1**	**5**	**7**	**10**	**15**
00	17.851 ± 0.09^aA^	17.861 ± 0.10^A^	17.882 ± 0.21^A^	17.902 ± 0.11^aA^	17.952 ± 0.32^aA^
4.238	19.860 ± 0.17^aB^	21.764 ± 0.45^bB^	29.961 ± 0.26^cB^	30.573 ± 0.55^cB^	32.840 ± 0.67^dB^
5.300	20.284 ± 0.67^aB^	23.463 ± 0.50^bB^	29.890 ± 0.15^cB^	32.571 ± 0.50^dB^	33.872 ± 0.60^dB^
10.600	22.763 ± 0.44^aC^	24.274 ± 0.65^aC^	30.853 ± 0.50^bB^	34.820 ± 0.53^cC^	36.091 ± 0.33^cC^

Note: Data are presented as mean ± SE of five fishes in each group, values with different alphabet superscript differ significantly (p < 0.05) between durations within concentration. Values with capital alphabet superscript differ significantly (p < 0.05) between concentrations within duration.

**Table 6.** Activity of catalase (unit/mg protein) in the liver of *C. punctatus* exposed to sub-lethal doses of atrazine for 1–15 days.

**Conc. mg·L^−1^**	**Exposure time (days)**				
	**1**	**5**	**7**	**10**	**15**
00	130.880 ± 0.47^aA^	130.910 ± 0.15^aA^	131.092 ± 0.58^aA^	131.531 ± 0.34^aA^	130.762 ± 0.66^aA^
4.238	165.122 ± 4.33^aB^	172.011 ± 4.25^aB^	165.430 ± 2.16^aB^	159.640 ± 3.12^aB^	150.322 ± 0.57^bB^
5.300	165.292 ± 2.71^aB^	175.604 ± 3.44^abB^	185.630 ± 3.22^bC^	180.361 ± 2.80^bC^	156.181 ± 3.42^aB^
10.600	171.981 ± 4.30^aB^	177.240 ± 3.11^abB^	235.192 ± 3.22^cD^	202.444 ± 3.11^dD^	164.282 ± 2.33^acB^

Note: Data are presented as mean ± SE of five fishes in each group, values with different alphabet superscript differ significantly (p < 0.05) between durations within concentration. Values with capital alphabet superscript differ significantly (p < 0.05) between concentrations within duration.

**Table 7.** Activity of glutathione reductase (nmol/mg protein) in the liver of *C. punctatus* exposed to sub-lethal doses of Atrazine for 1–15 days.

**Conc. mg·L^−1^**	**Exposure time (days)**				
	**1**	**5**	**7**	**10**	**15**
00	10.043 ± 0.57^aA^	10.050 ± 0.04^aA^	10.211 ± 0.033^aA^	10.060 ± 0.15^aA^	10.052 ± 0.38^aA^
4.238	10.814 ± 0.22^aA^	10.202 ± 0.51^aA^	10.401 ± 0.44^aA^	12.162 ± 0.61^aB^	12.911 ± 0.69^aB^
5.300	10.902 ± 0.67^aA^	11.013 ± 0.60^aA^	11.544 ± 0.96^aA^	13.842 ± 0.55^aB^	15.524 ± 0.96^bB^
10.600	10.602 ± 0.60^aA^	11.033 ± 0.64^aA^	12.023 ± 0.65^abA^	14.503 ± 1.1^bcC^	17.402 ± 0.40^cC^

Note: Data are presented as mean ± SE of 5 fishes in each group, values with different alphabet superscript differ significantly (p < 0.05) between durations within concentration. Values with capital alphabet superscript differ significantly (p < 0.05) between concentrations within duration.
